# Development of a highly sensitive and specific intact proviral DNA assay for HIV-1 subtype B and C

**DOI:** 10.1186/s12985-024-02300-6

**Published:** 2024-01-31

**Authors:** N. V. E. J. Buchholtz, M. M. Nühn, T. C. M. de Jong, T. A. T. Stienstra, K. Reddy, T. Ndung’u, Z. M. Ndhlovu, K. Fisher, S. Palmer, A. M. J. Wensing, J. Symons, M. Nijhuis

**Affiliations:** 1 Translational Virology, Department of Medical Microbiology, University Medical Center Utrecht, Heidelberglaan 100, 3584C Utrecht, The Netherlands; 2 Africa Health Research Institute (AHRI), Durban, South Africa; 3 HIV Pathogenesis Programme, The Doris Duke Medical Research Institute, University of KwaZulu-Natal, Durban, South Africa; 4 The Ragon Institute of Massachusetts General Hospital, Massachusetts Institute of Technology, Harvard University, Cambridge, MA 01238 USA; 5 Division of Infection and Immunity, University College London, London, UK; 6 Centre for Virus Research, The Westmead Institute for Medical Research, The University of Sydney, Sydney, NSW Australia; 7 ha, Faculty of Health Sciences, University of the Witwatersrand, Johannesburg, South Africa; 8 HIV Pathogenesis Research Unit, Faculty of Health Sciences, University of Witwatersrand, Johannesburg, South Africa

**Keywords:** HIV-1, IPDA, Subtype B and C, Intact-reservoir, Quantification, Global cure

## Abstract

**Introduction:**

HIV reservoir quantification is essential for evaluation of HIV curative strategies and may provide valuable insights about reservoir dynamics during antiretroviral therapy. The Intact Proviral DNA Assay (IPDA) provides the unique opportunity to quantify the intact and defective reservoir. The current IPDA is optimized for HIV-1 subtype B, the dominant subtype in resource-rich settings. However, subtype C is dominant in Sub-Saharan Africa, jointly accounting for around 60% of the pandemic. We developed an assay capable of quantifying intact and defective proviral HIV-1 DNA of subtype B and C.

**Methods:**

Primer and probe sequences were strategically positioned at conserved regions in *psi* and *env* and adapted to subtype B&C. In silico analysis of 752 subtype B and 697 subtype C near-full length genome sequences (nFGS) was performed to predict  the specificity and sensitivity. Gblocks were used to determine the limit of blank (LoB), limit of detection (LoD), and different annealing temperatures were tested to address impact of sequence variability.

**Results:**

The in silico analysis showed that the HIV-1 B&C IPDA correctly identified 100% of the intact subtype B, and 86% of the subtype C sequences. In contrast, the original IPDA identified 86% and 12% of these subtype B and C sequences as intact. Furthermore, the HIV-1 B&C IPDA correctly identified hypermutated (87% and 88%) and other defective sequences (73% and 66%) for subtype B and C with comparable specificity as the original IPDA for subtype B (59% and 63%). Subtype B cis-acting sequences were more frequently identified as intact by the HIV-1 B&C IPDA compared to the original IPDA (39% and 2%). The LoB for intact proviral DNA copies was 0, and the LoD for intact proviral DNA copies was 6 (> 95% certainty) at 60 °C. Quantification of 2–6 copies can be performed with > 80% certainty. Lowering the annealing temperature to 55 °C slightly lowered the specificity but prevented exclusion of samples with single mutations in the primer/probe region.

**Conclusions:**

We developed a robust and sensitive assay for the quantification of intact and defective HIV-1 subtype B and C proviral DNA, making this a suitable tool to monitor the impact of (large-scale) curative interventions.

**Supplementary Information:**

The online version contains supplementary material available at 10.1186/s12985-024-02300-6.

## Introduction

Human immunodeficiency virus type 1 (HIV-1) persists within latently infected cells in people with HIV (PWH), despite lifelong effective antiretroviral therapy (ART) [1]. In search for a cure, developing accurate assays that quantify and characterize the persistent HIV reservoir is crucial. Most HIV-infected cells contain defective proviruses, and the ratio of defective proviral DNA compared to intact depends on treatment duration [2–4]. These defective proviruses are in general characterized by large deletions and hypermutations [5]. A challenge in the quantification of the HIV reservoir is to accurately detect and quantify the replication competent viral reservoir that may cause viral rebound when reactivated. The quantitative viral outgrowth assay (QVOA) does not induce all cells harboring replication competent HIV after one round of activation, resulting in an underestimation of the true replication competent reservoir [6–9]. Moreover, this assay is labor intensive and expensive [10]. Alternatively, total HIV DNA measured via a single-probe polymerase chain reaction (PCR) generally results in an overestimation of the replication competent reservoir [10].

In 2019, Bruner et al*.* developed the Intact Proviral DNA Assay (IPDA), which is a multiplex droplet digital PCR (ddPCR). Two HIV-1 regions are targeted simultaneously by two primer and probe subsets; the packaging region (ψ/*psi)* at the 5′ end, and the REV response element (RRE) region of the envelope (*env)* gene at the 3′ end [5, 11]. Additionally, defective hypermutated sequences in the *env* target are excluded from intact sequences via an unlabeled probe to prevent false positive detection of hypermutated sequences at this 3′ signal [5, 12]. Bruner et al*.* confirmed via a direct comparison of IPDA results with near-full-length genome sequencing (nFGS) data on subtype B that this assay can accurately quantify and distinguish intact, which are potentially replication competent, from defective proviruses [5]. Moreover, levels of intact proviruses correlate with the levels of inducible proviruses in the QVOA but are about 50-fold higher [5, 13]. Nevertheless, several concerns regarding the impact of viral polymorphisms on the IPDA have been raised in other studies [12, 14]. A 28% failure rate due to viral polymorphisms has been described for the IPDA on HIV-1 subtype B [12].

Furthermore, the original IPDA has been developed to quantify HIV-1 subtype B, which is predominant in resource-rich settings. To increase equity in HIV treatment and cure, it is important to develop quantification assays with broader subtype-specificity [15]. Subtype C is the most dominant variant worldwide with a prevalence of ~46%, predominantly present in Sub-Saharan Africa, India, and Ethiopia [16, 17]. Because of sequence diversity across subtypes [18, 19], primers and probes used in the IPDA need careful revision. In this paper, we describe the development of a sensitive HIV-1 subtype B&C IPDA and provide an overview of its challenges and limitations.

## Methods

### Primers, probes and Gblock design

Primer and probe sequences were adapted based on the intact genomes from Los Alamos National Laboratory HIV Sequence Compendium 2018 (https://www.hiv.lanl.gov). All primers, probes, and Gblocks (double-stranded DNA fragments) used within this paper were designed and ordered at IDT (Table 1, Additional file 1: Table S1). RPP30 primers and probes used in this study were identical to the original IPDA described by Bruner et al*. *[5, 12] (Table 1). *Psi* and *env* primers for both subtypes were combined in one PCR master mix for the B&C IPDA.

**Table 1:** Overview of primers and probes for the subtype B&C IPDA

Primer	Sequence	Position HXB2
*psi* forward	TCTCGACGCAGGACTCG	684–700
*psi* probe	/56-FAM/CTCTCTCCT/ZEN/TCTAGCCTC/3IABkFQ/	772–789
*psi* reverse subtype B	TACTGACGCTCTCGCACC	793–810
*psi* reverse subtype C	TATTGACGCTCTCGCACC	793–810
*env* forward subtype B	AGTGGTGCAGAGAGAAAAAAGAGC	7736–7759
*env* forward subtype C	AGTGGTGGAGAGAGAAAAAAGAGC	7736–7759
*env* probe	/5HEX/CCTTGGGTT/ZEN/CTTGGGAGC/3IABkFQ/	7781–7798
*env* hypermutation probe	/5IABkFQ/CCTTAGGTTCTTAGGAGC/3IABkFQ/	7781–7798
*env* reverse	GTCTGGCCTGTACCGTCAGC	7851–7832
RPP30 forward 1	GATTTGGACCTGCGAGCG	n/a
RPP30 reverse 1	GCGGCTGTCTCCACAAGT	n/a
RPP30 probe	/5HEX/CTGACCTGA/ZEN/AGGCTCT/3IABkFQ/	n/a
RPP30 forward 2	CCATTTGCTGCTCCTTGGG	n/a
RPP30 reverse 2	CATGCAAAGGAGGAAGCCG	n/a
RPP30 probe 2	/56-FAM/AAGGAGCAA/ZEN/GGTTCTATTGTAG/3IABkFQ/	n/a

The red nucleotides mark the differences between subtype B and C, and the *env* probe with the hypermutation probe

### Identification of intact and defective proviral DNA by the B&C IPDA primers and probes

To predict correct identification of defective and intact proviral DNA, previously described, characterized, and annotated near-full-length subtype B (n = 2125) [20–22] and subtype C sequences (n = 697) [23, 24] were mapped to the HXB2 reference strain using SeqScape Software v4.0. Sample collection and sequencing started in 2017 and was completed in 2021. Subtype B sequences were obtained by full-length individual proviral sequencing (FLIPS) [21, 25] of 25 participants on long-term ART [20–22]. Subtype C sequences were obtained from 24 participants who were longitudinally investigated during both acute and chronic infection within the FRESH cohort [23, 24]. Genetically intact sequences were categorized as those lacking multiple defects using comparable pipelines in the respective cohorts. Exclusion criteria contained large deletions, hypermutations, stop codons, cis-acting sequences, and frameshift mutations. The exact pipelines per subtype are explained in more detail in Additional file 1: Fig. S1. The above mentioned studies have been reviewed by ethical committee. All participants provided written consent. Intact, hypermutated, proviral genomes with mutations in the cis-acting region and other defective proviral genomes were analyzed separately. Our subtype B&C IPDA primer/probe subsets were compared to the sequences of the subtype B and C sequences described above. Cutoffs for primer and probe mismatches were applied as previously described by Gaebler et al*.* [26]. A maximum of one probe mismatch and four primer mismatches was permitted. Primers were subdivided in a 5′, middle and 3′ region and 3 mismatches in the 5′ and 1 mismatch in the 3′ end of the primers was allowed. Subsequently, the predicted success of the binding of either the *psi* and/or *env* primers and probe sets to a specific sequence were combined to predict whether the IPDA would identify this sequence as intact or defective. Binding of both primer and probe sets represented an intact sequence, binding of just one set represented a defective sequence, binding of neither resulted in “inferred lack of signal”.

### IPDA

Proviral DNA was quantified using a multiplex ddPCR, targeting parts of the Ψ (*psi*) and *env* region, using the primer sets described above. The RPP30 cellular gene was used to measure the analyzed number of cells, and the DNA shearing index (DSI), being an indication of DNA fragmentation [5]. DNA from PBMCs of HIV-negative donors was used as a DNA template control, J-Lat full-length clones 15.4 cells (NIH AIDS Reagent Program) as a HIV-positive control, and water as a no template control. Gblocks were used to determine the sensitivity and specificity of the assay, to specify shearing corrections, and to test the sensitivity at different annealing temperatures. Cycle conditions were performed according to manufacturer’s protocol (Biorad), except for annealing temperature variations of 60 °C, 58 °C, 55 °C and 53 °C (Table 2). Quantasoft version 1.7.4. was used for results analysis. Positive and negative droplets were discriminated by manual thresholding [27].

**Table 2:** Thermal cycle protocol

Cycling step	Temperature °C	Time	Ramp rate	#cycles
Enzyme activation	95	10 min	~ 2 °C/sec	1
Denaturation	94	30 s		40
Annealing/extension	53, 55, 58 or 60	1 min		
Enzyme deactivation	98	10 min		1

### DNA isolation

Genomic DNA was isolated using the DNeasy Blood and tissue kit (Qiagen), according to manufacturer’s guidelines.

### Quantification and statistical analyses

Statistical analyses were performed using GraphPad Prism v7.0 (GraphPad Software, San Diego, California, USA) and R 4.1.2. Statistical tests are indicated within the figure legends, *p*-values of < 0.05 were considered significant. Graphs were composed using Excel (Microsoft Office), Biorender.com, GraphPad Prism v7.0 (GraphPad Software, San Diego, California, USA) and R 4.1.2.

## Results

### Design of primer and probe sequences

To explore the options of an IPDA applicable for both subtype B and C, the Los Alamos database (https://www.hiv.lanl.gov) was consulted which offers a comprehensive overview of sequence variability. A conserved region across subtypes downstream of the original *psi* primers and probe could be found. The conservation of new IPDA primer and probe sets between subtypes was analyzed by using near-complete genomes from the Los Alamos National Laboratory HIV Sequence Compendium by checking whether the sequences had a 100% match. In total 148 unique sequences from different subtypes as published in the compendia 2014, 2016, and 2020 were analyzed for homology to both primer and probe sets.

**Fig. 1:**
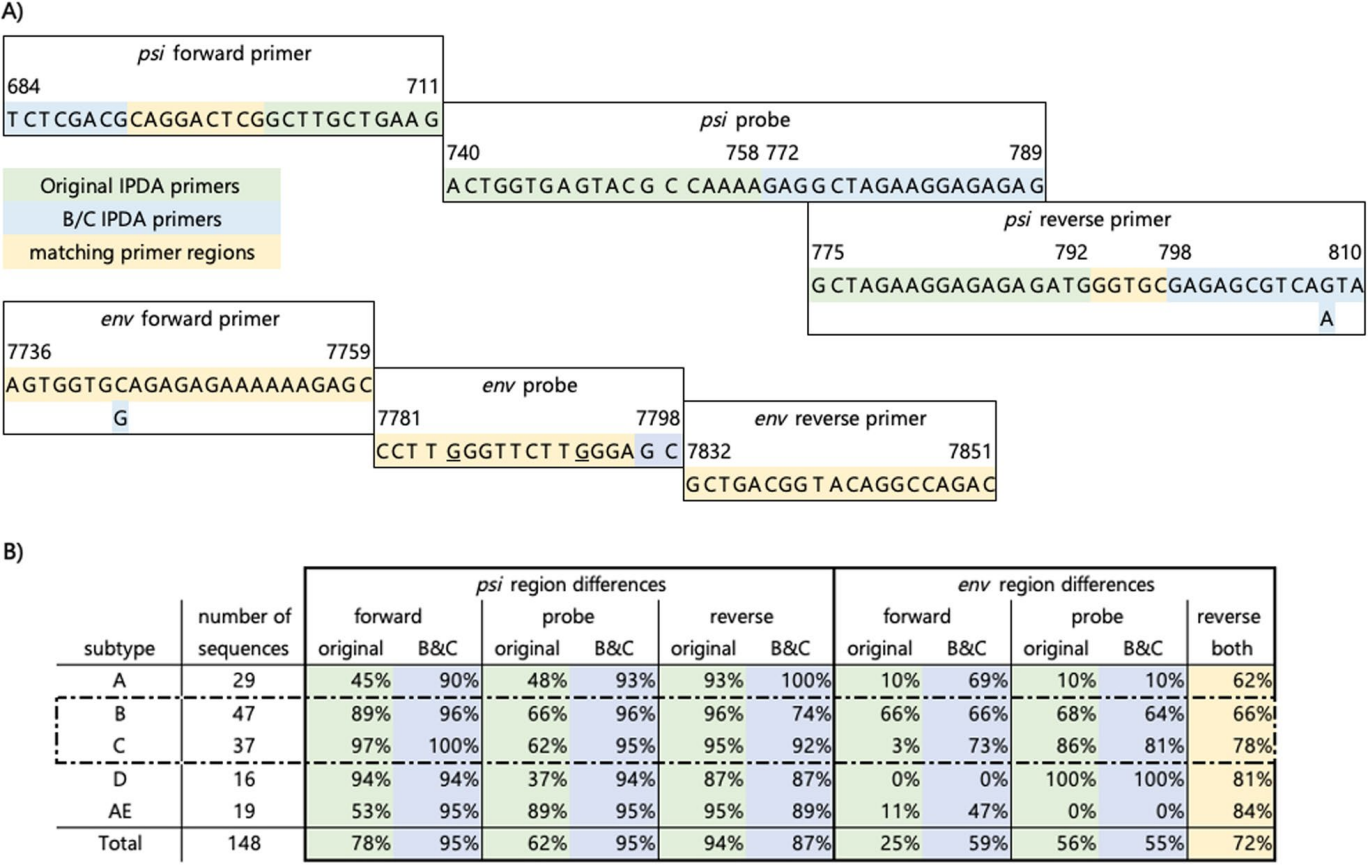
Comparison of the original and subtype B&C IPDA primer and probe subsets. **A** Overview of the target regions of the *psi* and *env* primers and probes. The original target sequences are indicated in green, the subtype B&C IPDA sequences in blue, overlapping sequences in yellow. Nucleotide positions relative to the HXB2 sequence are indicated. The two nucleotide changes in the hypermutated probe for *env* are underlined. **B** Frequency of homologous sequences within the Los Alamos compendium 2014, 2016 and 2020 for the original and the subtype B&C IPDA primers and probes

Overall, the *psi* target region was moved to a more conserved region across subtypes and the *env* primer/probe set was slightly adapted. The *psi* reverse primer and *env* forward primer both contain 1 nucleotide difference between subtype B and subtype C. Both subtype specific primer/probe sets were combined in the same mixture for the B&C IPDA (Fig. 1A, B; Additional file 1: Figure S2).

### Classification of intact and defective proviral DNA by the B&C IPDA primers and probes

To assess the performance of our primers and probes on HIV sequences from PWH, we received 697 near full length subtype C and 2125 near full length subtype B sequences for in silico analysis. These sequences have been previously annotated as “intact”, “hypermutated”, “other defective”, and “cisacting”. The “other defective” sequences have large deletions, inversions, stop codons or frameshifts.

**Fig. 2:**
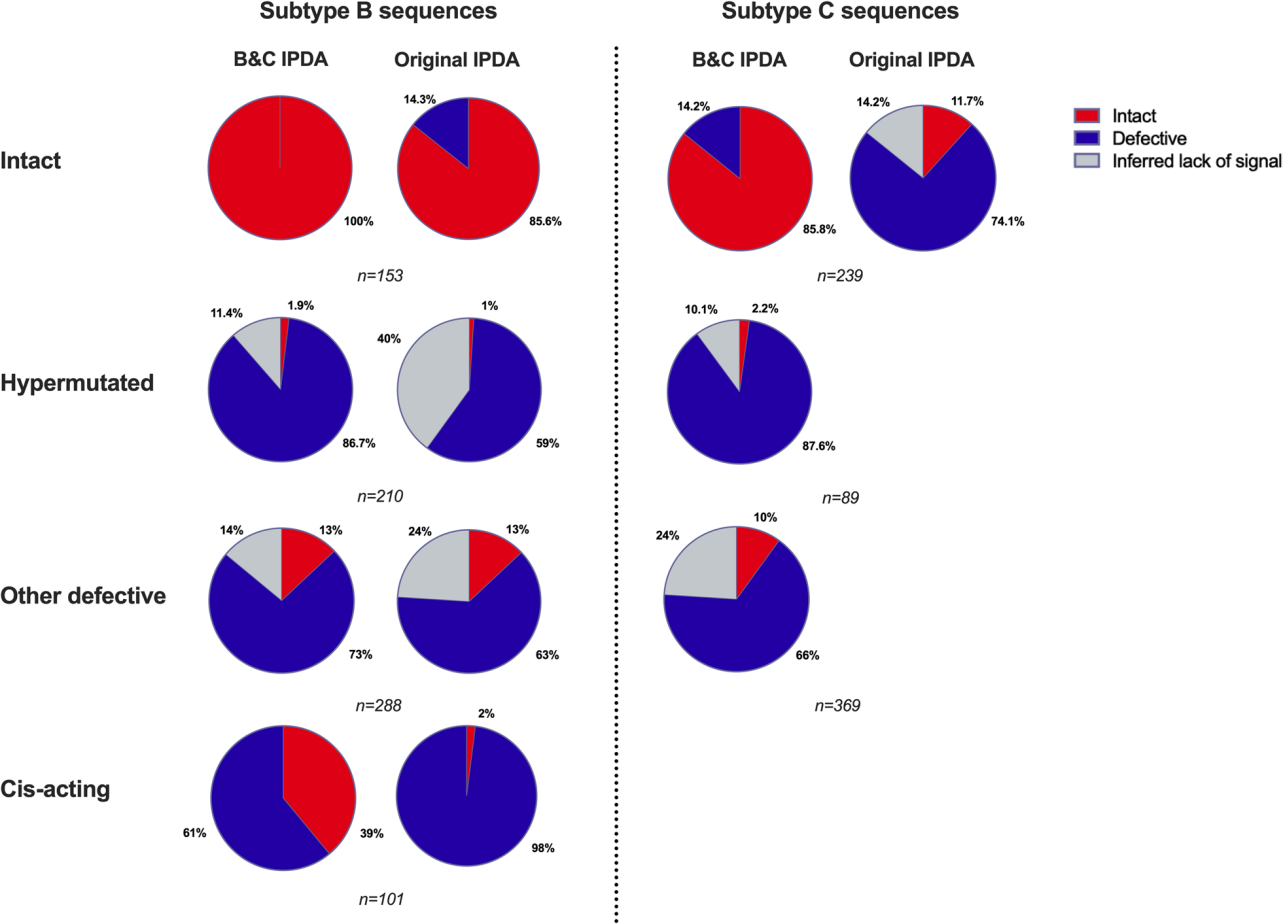
In silico analysis of the annotation of near-full length HIV-1 subtype B and subtype C sequences by the original and subtype B&C IPDA. 752 Subtype B and 697 subtype C sequences were analyzed and classified as intact, hypermutated, other defective and cis-acting sequences according to their annotation after sequencing. Frequency of these sequences predicted to be intact, defective, or inferred lack of signal by the IPDA are depicted. Because of large mismatches of the original IPDA primers and probes for intact subtype C sequences, no analyses were shown for defective subtype C sequences with the original IPDA. These details can be found in Additional file 1: Table S2

Among the 2125 subtype B sequences, 752 were randomly chosen, ensuring a fair distribution of sequences from each participant. This selection aimed to achieve a more balanced and comparable distribution of subtype B and C sequences for the various annotations. We found that 100% of intact subtype B and 85.8% of intact subtype C sequences were correctly classified by our new primer/probe set (Fig. 2 and Additional file 1: Table S2). In contrast, only 11.7% of the intact subtype C sequences were correctly predicted to be intact according to the original IPDA (Fig. 2 and Additional file 1: Table S2), largely due to sequence variation in the *psi* probe region. Furthermore, the B&C IPDA correctly classified subtype B (86.7%) and subtype C (87.6%) hypermutated sequences as defective, while just 1.9% and 2.2% were falsely classified as intact (respectively). Within the class of “other defectives”, 73% and 66% of the respectively subtype B and subtype C sequences were correctly identified as defective, and 13% and 10% falsely as intact (Fig. 2 and Additional file 1: Table S2). When comparing this to the performance of the original IPDA for subtype B sequences, the percentage of sequences falsely identified as intact was similar between the original and the new B&C IPDA (Fig. 2 and Additional file 1: Table S2). In contrast to the original IPDA, the new *psi* target region of the B&C IPDA does not include the Major Splice Donor Site (MSD) [8, 28, 29]. Cis-acting sequences hold mutations in any of the 4 stem loops of the packaging signal at HXB2 nucleotide position 695–810 and/or MSD site between nucleotides 744–745, while the rest of the genome is mostly intact [20–22]. The cis-acting sequences represented 4.8% of the subtype B sequences (Additional file 1: Table S2), of which 39% was falsely identified as intact by the B&C IPDA, representing 1.9% of the subtype B sequence cohort. With the original IPDA only 2% of the cis-acting sequences for subtype B were falsely identified as intact. Within the subtype C cohort, 1.4% of the sequences had deletions or insertions within the packaging signal, but these represent larger defects compared to the cis-acting subtype B sequences and did not have any specific mutations in the MSD site.

### IPDA failures

PCR assays targeting the HIV genome are subject to variation in amplification efficiency due to polymorphisms in the viral sequence [11–13, 30]. Viral diversity is common and may lead to failure of amplification and thus underestimation of the (intact) proviral reservoir, especially in regions that are highly variable like the *env* region. In particular, mismatches in probe regions have a major effect on quantification [26, 31]. As suggested by Kinloch et al*.* and Falcinelli et al*. *[12, 13], the issue of these typical failures in *env*, can be resolved by decreasing the PCRs annealing temperature. To explore the best annealing temperature for the B&C IPDA, several Gblocks containing either an intact sequence matching the probe sequence, or single/double G-A mutations at the 5th or 13th nucleotide of the *env* (hypermutation) probe region, designed by Bruner et al.[5], were tested at annealing temperatures of 60 °C, 58 °C, 55 °C, and 53 °C (Additional file 1: Table S1 and Table 3). These single nucleotide changes represent the viral diversity in PWH, whereas the double mutations represent a hypermutated, and thus defective sequence. We demonstrated that at annealing temperatures of 60 °C or 58 °C only exact matching sequences could be amplified and thus quantified. At 55 °C sequences with one mutation in the target region could be correctly quantified. Decreasing the annealing temperature to 53 °C leads to detection of the hypermutated Gblock by the *env* probe, indicating that a hypermutated sequence may be quantified as intact, resulting in a loss of the specificity needed to reliably distinguish intact from defective hypermutated sequences (Table 3).

**Table 3:** Overview of the IPDA at different annealing temperatures

		Number of detected copies			
Gblock (input 1000 copies)		Annealing temperature			
Sequence at probe region	Description	60 °C	58 °C	55 °C	53 °C
CCTTGGGTTCTTGGGAGC	Env intact matching probe	1293.6	1238.6	1001.0	1273.1
CCTT**A**GGTTCTTGGGAGC	Env G-A mutation 5th nucleotide	2.0	2.8	1021.4	1372.1
CCTTGGGTTCTT**A**GGAGC	Env G-A mutation 13th nucleotide	0.6	0.0	840.4	1177.7
CCTT**A**GGTTCTT**A**GGAGC	Env G-A mutation 5th and 13th nucleotide	0.0	0.9	0.4	2.3

Gblock copies containing none, one or two mutations in the *env* probe were used to test the specificity of detecting only intact *env* copies. The Gblock containing the intact sequence does match the probe sequence. The Gblock with two mutations matches the unlabeled hypermutation probe

### Limit of blank (LoB)

A limitation of the ddPCR are the false positive signals, which can arise from random, assay-independent artefacts [32]. To test the specificity of the B&C assay, the limit of blank (LoB) was determined [33]. As a negative control, PBMCs of HIV-negative donors were analyzed. At 60 °C (n = 81) single positive droplets were detected with a mean of 0.12 copies, and a standard deviation of 0.48, leading to an LoB of 0.90 copies (Table 4) [34]. At 55 °C (n = 24), the LoB was determined at 1.66 copies (Table 4). The false positive droplets were never detected in the intact channel, resulting in a LoB of intact copies of 0.

**Table 4:** Overview of the LoB and LoD

Limit of Blank (LoB) on donor PBMCs
LoB = mean_blank_ + 1.645 × (SD blank)
LoB 60 °C = 0.12 + 1.645 × 0.48 = 0.90
LoB 55 °C = 0.34 + 1.645 × 0.80 = 1.66

Limit of detection (LoD) on Gblocks					
Copy input	Mean copy output	Standard deviation	Number of repeats	% Positive signals	95% CI
*60 °C*					
6	6.38	3.68	40	97.5	5.24–7.52
5	4.15	2.99	40	92.9	3.25–5.05
4	3.50	2.36	42	90.0	2.77–4.23
3	3.21	2.22	41	95.1	2.53–3.89
2	1.91	1.63	42	83.3	1.42–2.40
1	1.11	1.19	44	61.4	0.76–1.46
*55 °C*					
5	3.56	2.37	44	95.5	2.86–4.26
4	2.46	1.77	44	90.9	1.93–2.98
3	2.28	1.97	44	84.1	1.70–2.86
LoD = LoB + 1.645 × (SD sample 95% positivity)					
LoD 60 °C = 0.90 + (1.645 × 3.68) = 6.9					
LoD 55 °C = 1.66 + (1.645 × 2.37) = 5.6					

The LoB has been determined by using DNA derived from PBMCs of HIV-negative donors. The LoD and the 95% CI of an annealing temperature of 60 °C and 55 °C are calculated by running replicates of Gblocks containing the specific *psi* and *env* sequences for 1–6 copies. For 0 copies, the LoB values of DNA derived from PBMCs of HIV-negative donors are depicted

### Limit of detection (LoD) of the assay and precision

Only a relatively small fraction of cells contains HIV DNA, making it important to determine the limit of detection (LoD) [33], representing the sensitivity. The LoD was determined using Gblocks containing both intact *psi* and *env* primer/probe sequences (Additional file 1: Table S1). These Gblocks were diluted ranging from 1 to 6 copies (Table 4). For every dilution at least 40 replicates were performed, the assay is considered as true positive with a positive signal in 95% of the replicates [34]. We demonstrated that 6 copies input showed a positive signal in > 95% of the replicates (Table 4). Subsequently, the LoD can be calculated with the standard deviation of these 6 copies [34] which together with the LoB resulted in a LoD of 7 copies for single/defective copies (see Table 4 for formula). Additionally, detection of 1–5 copies is not unreliable, because this still provides a certainty of 61.4–92.9% (Table 4). Moreover, the 95% confidence interval (CI) of the detected copies matched with the input numbers, representing precise detection. As indicated above, the LoB is around 0 for the double positive droplets, indicating that the LoD for the intact copies would be 6. The same experiments for the LoD were performed with an annealing temperature at 55 °C. Within these assays, the LOD was determined at 4 copies for intact and 6 for defective copies (Table 4).

### Shearing calculations

**Fig. 3:**
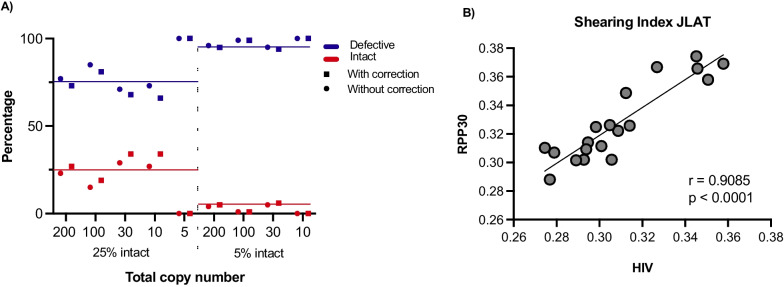
Mix of different fractions of defective and intact Gblocks. **A** The defective copies are indicated in blue, the intact copies in red. The expected percentage of copies is indicated with a line. Squares represent the copy numbers with shearing correction, the circles without shearing correction. Total number of copies used as input are indicated on the x-axis. **B** The shearing index measured for RPP30 and HIV (*psi/env*) on Jlat DNA extractions (n = 19) correlated significantly (*p* < 0.0001)

To correctly quantify the reservoir, a correction for the shearing of the DNA as generated during processing is needed. Therefore, two regions within the RPP30 cellular gene that are 7 kb apart, which is the same distance as the *psi* and *env* region, are quantified in parallel to determine the DSI [5]. This DSI can be used to correct the impact of shearing on the number of intact HIV copies as shown in detail in Additional file 1: Fig. S3. Next, we tested the accuracy, with and without shearing correction, of the B&C IPDA, with mixtures of the double mutation env Gblock and 25% to 5% of the intact Gblock, Additional file 1: Table S1. We then spiked low copy numbers, ranging from 5 to 200 copies, of these different mixes in a background of PBMCs and used the B&C IPDA to quantify the level of intact and defective provirus. The calculated percentage of intact and defective copies were as expected and shearing correction slightly improved the quantification (Fig. 3A). When measuring below the LoD, the 5 copy-sample, no intact copies were reported. Since our Gblocks contained less than 7 kb distance we also tested shearing in J-Lat, and found in accordance with Bruner et al*.*, that the shearing levels calculated on RPP30 and *psi*/*env* (HIV) within J-Lat cells were comparable (Fig. 3B) [5]. This confirms that the shearing index can be subsequently used to correct the number of intact HIV copies.

### Clinical samples

**Fig. 4:**
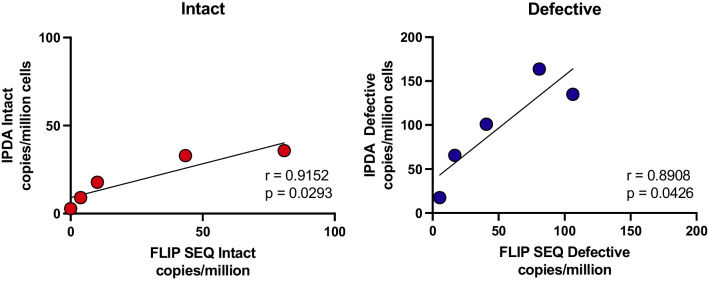
Correlation between the IPDA and FLIP sequencing data. The intact and defective reservoir correlated significantly with the FLIP analysis for the 5 chronically treated PWH subtype C (*p* < 0.05)

To assess the applicability of the new primers and probes as a diagnostic tool, we conducted a validation assay using clinical samples. Five subtype C samples from chronically treated PWH, previously analyzed using FLIPS [23, 24], were subjected to the new subtype B&C IPDA. The resulting graphs indicate significant correlations for the FLIPS data with both the intact and the defective reservoir, *p* < 0.05 (Fig. 4).

## Discussion

Accurate quantification of the HIV reservoir and effective monitoring of reservoir size during antiretroviral therapy and/or cure interventions require specific and sensitive assays that can be conducted with a limited number of cells, are not labor intensive, and affordable [35]. The IPDA developed by Bruner et al*.* meets all these requirements but is designed for subtype B sequences [5]. Optimizing this assay for subtypes more prevalent in areas in which the need for a cure is high, would be highly advantageous [15]. We optimized the original IPDA by moving the target region of the *psi* primers and probes and included minor changes in the *env* primers and probes. We showed via in silico analyses with nFGS data of two cohorts covering subtype B and subtype C sequences, a correct identification of intact subtype C (100%) and subtype B (86%). We determined that the assay could detect 6 intact copies and 7 defective copies at an annealing temperature of 60 °C with 95% certainty. Furthermore, 2–5 copies can still be quantified with > 80% certainty. When lowering the annealing temperature, the sensitivity for the detection of intact sequences is also increased. Lastly, we showed that the assay could precisely quantify mixtures of different proportions of defective and intact sequences, especially when applying shearing corrections.

With the in silico analysis of near-full length sequences the sensitivity and specificity of the B&C IPDA were compared to the original IPDA. We observed a higher sensitivity of the B&C IPDA for FLIPS-annotated intact subtype C, as opposed to the original IPDA. Specifically, we observed that 86% of the intact sequences were classified as intact by the B&C IPDA versus just 12% by the original IPDA. When comparing the performance of both assays for subtype B sequences, the B&C IPDA demonstrates a higher accuracy in labeling FLIPS-annotated intact, hypermutated and other defective sequences. On the other hand, the B&C IPDA identifies 39% of the subtype B cis-acting sequences as intact, representing 1.9% of the total subtype B sequence cohort, because the *psi* primer–probe set is not located in the MSD site. For the original IPDA, in which the *psi* primer–probe set does target the MSD site, this overestimation only occurs in 2% of the subtype B cis-acting sequences. We realize that overestimation of the intact reservoir may pose a problem. However, the usage of a general subtype C *psi* probe including the MSD site is hardly possible, because of subtype C sequence variations within this particular region. An alternative might be the development of cohort-specific probes as recently presented for subtype A and D [36]. However, this lowers feasibility, making it especially difficult when limited resources are available.

On the other hand, underestimation of the size of the intact reservoir may pose a greater risk than an overestimation of the intact reservoir, especially during curative interventions. Kinloch et al*.* discussed that the natural HIV sequence variation between and within participants and mismatch of one primer and/or probe target may lead to the underestimation of the intact reservoir by the original IPDA [11, 12]. By lowering the annealing temperature, it has been suggested that this underestimation issue might be solved [12, 13]. Indeed, we showed that by lowering the annealing temperature to 55 °C, proviruses with single nucleotide changes in the primer/probe region could be detected, which would have been missed at annealing temperature of 60 °C. However, lowering the annealing temperature resulted in a lower specificity illustrated by an increase of the LoB at 55 °C. Depending on the research questions, one could choose for the highest specificity (60 °C) or the better chance of addressing minor HIV polymorphisms (55 °C). Additionally, it is still important to critically evaluate whether ddPCR plots show indications of mismatches of primers and/or probes to the HIV sequences within a specific individual. In this case, exclusion of samples or adjustment of individual-specific primers and/or probes might be needed [11, 12, 30].

Within most intervention studies, frequent occurrence of low copy numbers and the limited availability of sample occurs, which might consequently lead to a reservoir size below the LoD. This does not automatically indicate the absence of HIV copies, if on forehand determined and consequently used cut-off values are applied, for which we provide the following guidelines. Typically, HIV reservoirs are being reported as the number of HIV copies per million PBMC or CD4^+^ T cells, Additional file 1: Fig. S3. We advise to use a minimum of 100.000 cells for the analyses, so that the observed copy number will not be multiplied by more than 10 to reach the reported number of copies per million cells. If no copies are detected in 100.000 cells, we recommend using left censoring [30, 37] and advise to report and visualize these copy numbers differently within graphs by for example showing an open symbol versus a closed symbol. Altogether, our HIV-1 subtype B&C IPDA has a lower threshold of 6 intact proviral DNA copies and 7 *psi* and *env* copies at an annealing temperature of 60 °C, for 55 °C this is 4 intact copies and 6 *psi* and *env* copies. In case these copy numbers are not reached we recommend testing at least 100.000 cells or otherwise consider the sample not suitable for quantification. Lastly, we advise to exclude samples with a DSI of > 50%, because of bad sample quality.

Our study acknowledges certain limitations, such as the analysis of subtype B and C sequences via slightly different pipelines by different research institutes as shown in Additional file 1: Figure S1. Moreover, this analysis was performed on nFGS rather than full-length sequences, which could potentially overcall the intact reservoir. Additionally, most optimizations were performed on Gblocks instead of clinical samples, although we did show a correlation between the nFGS data and the B&C IPDA within 5 subtype C individuals for both defective and intact proviruses. Nevertheless, we used the B&C IPDA on multiple additional clinical samples from PWH with subtype B or subtype C and were able to get insight in the relationship between (intact) proviral DNA and the activity of the viral reservoir. However, no nFGS data is available for these individuals, which limits the direct comparison of the IPDA results with the annotation according to the bioinformatic tools.

Even though our B&C IPDA was optimized for the quantification of subtype B and C, other subtypes such as D, F, H, J and K do not show any sequence variation, or just one mismatch, for our target primer/probes sets according to the Los Alamos National Laboratory HIV Sequence Compendium 2014, 2016 and 2019. We tested viral cultures of clinical isolates of subtype D (n = 1) and F (n = 1) [38], and detected intact proviral DNA with the B&C IPDA for both subtypes. However, since we do not have any additional clinical samples or nFGS of these subtypes, further work on these subtypes is required. Unfortunately, viral cultures of clinical isolates of subtype A and circulation recombinant form (CRF) AE showed that the B/C IPDA could not be used for the detection of these subtypes because of the inability to detect the *env* region. This might be caused by the two mismatches within the *env* probe according to the Los Alamos National Laboratory HIV Sequence Compendia, of which one is a G-A mutation of the 13th nucleotide of this probe. This mutation corresponds to nucleotide differences between the *env* probe and the *env* hypermutation probe and might therefore contribute to the fact that the *env* probe is less likely to bind to subtype A and CRF AE sequences.

The field of intact HIV quantification using PCR based assays is evolving rapidly. Multiplex ddPCR assays with 4 or 5 fluorescent targets have been developed [27, 33], of which one is even optimized to quantify subtype A, C, D and CRF01_AE reservoirs [39]. Moreover, other methods such as the quadruplex PCR (Q4PCR), also target multiple sub-genomic regions [26]. The obvious advantage of these assays is a better distinction of the intact reservoir from the defective reservoir. However, the downside may be the increased variation accompanied with the use of more primer and probes sets [40], because of the previously discussed occurrence of polymorphisms. Moreover, an advantage of the 2-target ddPCR over an assay with more targets is that fewer cells are needed. It is easier to analyze, less expensive, and less labor-intensive, making it, once again, more suitable for implementation in settings with limited resources.

## Conclusions

In conclusion, the HIV-1 B&C IPDA ensures fast, sensitive, and specific quantification of the intact and defective subtype B and C viral reservoir in PWH, which makes it a suitable candidate for the versatile monitoring of HIV cure interventions in resource-rich settings and in large regions of Sub-Saharan Africa.

### Supplementary Information

**Additional file 1**. **Table S1**: Overview of Gblock sequences. **Table S2**: In silico analysis of subtype B and subtype C sequences for the original and subtype B&C IPDA. **Figure S1**: Overview of the pipelines to classify intact sequences for both subtypes. **Figure S2**: Detection efficiency of different subtype primers. **Figure S3**: Calculation example.

## Data Availability

Subtype B sequences which support the findings of this study have been uploaded to GenBank under accession numbers KY778264–KY778681, KY766150–KY766212, MW754554–MW754712, MZ080627–MZ081008, MN466964–MN467397, MZ922480–MZ923010, MZ962316, OL872744–OL873105, and OP700895-OP701628. Of subtype C sequences, 292 have been deposited in GenBank under accession numbers MK643536–MK643827. The remaining subtype C sequences will be published soon.

## Abbreviations

ART: Antiretroviral therapy ART
CI: Confidence interval
DSI: DNA searing index
*env*: Envelope
FLIPS: Full-length individual proviral sequencing
HIV-1: Human immunodeficiency virus type 1
IPDA: Intact proviral DNA assay
LoB: Limit of blank
LoD: Limit of detection
MSD: Major splice donor site
nFGS: Near-full length genome sequences
ψ/*psi*: Packaging region
PWH: People with HIV
PCR: Polymerase chain reaction
Q4PCR: Quadruplex PCR
QVOA: Quantitative viral outgrowth assay
RRE: REV response element
