# Supplementary material for: Development of a highly sensitive and specific intact proviral DNA assay for HIV-1 subtype B and C
Source: Virol J. 2024 Jan 31;21:36. doi: 10.1186/s12985-024-02300-6 (PMC10832250; doi:10.1186/s12985-024-02300-6)
Supplement: Supplementary file 1 — Additional file 1. Table S1: Overview of Gblock sequences. Table S2: In silico analysis of subtype B and subtype C sequences for the original and subtype B&C IPDA. Figure S1: Overview of the pipelines to classify intact sequences for both subtypes. Figure S2: Detection efficiency of different subtype primers. Figure S3: Calculation example. [file 12985_2024_2300_MOESM1_ESM.docx]

**Supporting information**

**Table S1. Overview of Gblock sequences**

| **Gblock** | **Sequence** |
| --- | --- |
| Subtype B *psi* + *env* + RPP30 + LTR | TGCTTAAGCCTCAATAAAGCTTGCCTTGAGTGCTTCAAGTAGTGTGTGCCCGTCTGTTGTGTGACTCTGGTAACTAGAGATCCCTCAGACCAGCTCTCTCGACGCAGGACTCGGCTTGCTGAAGCGCGCACGGCAAGAGGCGAGGGGCGGCGACTGGTGAGTACGCCAAAAATTTTGACTAGCGGAGGCTAGAAGGAGAGAGATGGGTGCGAGAGCGTCAGTATTAAGAGAAGAGTGGTGCAGAGAGAAAAAAGAGCAGTGGGAATAGGAGCTTTGTTCCTTGGGTTCTTGGGAGCAGCAGGAAGCACTATGGGCGCAGCCTCAATGACGCTGACGGTACAGGCCAGACAATTATTGCAGATTTGGACCTGCGAGCGGGGTTCTGACCTGAAGGCTCTGCGCGGACTTGTGGAGACAGCCGCTCACCTCTTACCATTTGCTGCTCCTTGGGAGGGAAAGGAGCAAGGTTCTATTGTAGCATAAAAGTTTCTACCTCTGTTTCGGCTTCCTCCTTTGCATGCTCTG |
| Subtype C *psi* + *env* + RPP30 + LTR | TGCTTAAGCCTCAATAAAGCTTGCCTTGAGTGCTTCAAGTAGTGTGTGCCCGTCTGTTGTGTGACTCTGGTAACTAGAGATCCCTCAGACCAGCTCTCTCGACGCAGGACTCGGCTTGCTGAAGCGCGCACGGCAAGAGGCGAGGGGCGGCGACTGGTGAGTACGCCAAAAATTTTGACTAGCGGAGGCTAGAAGGAGAGAGATGGGTGCGAGAGCGTCAATATTAAGAGAAGAGTGGTGGAGAGAGAAAAAAGAGCAGTGGGAATAGGAGCTTTGTTCCTTGGGTTCTTGGGAGCAGCAGGAAGCACTATGGGCGCAGCCTCAATGACGCTGACGGTACAGGCCAGACAATTATTGCAGATTTGGACCTGCGAGCGGGGTTCTGACCTGAAGGCTCTGCGCGGACTTGTGGAGACAGCCGCTCACCTCTTACCATTTGCTGCTCCTTGGGAGGGAAAGGAGCAAGGTTCTATTGTAGCATAAAAGTTTCTACCTCTGTTTCGGCTTCCTCCTTTGCATGCTCTG |
| Subtype C *psi* + intact *env* | CCCACTGCTTAAGCCTCAATAAAGCTTGCCTTGAGTGCTTCAAGTAGTGTGTGCCCGTCTGTTGTGTGACTCTGGTAACTAGAGATCCCTCAGACCAGAAGAGTGGTGCAGAGAGAAAAAAGAGCAGTGGGAATAGGAGCTTTGTTCCTTGGGTTCTTGGGAGCAGCAGGAAGCACTATGGGCGCAGCCTCAATGACGCTGACGGTACAGGCCAGACAATTATTGTC |
| Subtype C *psi* + single mutation position 5 *env* | CCCACTGCTTAAGCCTCAATAAAGCTTGCCTTGAGTGCTTCAAGTAGTGTGTGCCCGTCTGTTGTGTGACTCTGGTAACTAGAGATCCCTCAGACCAGAAGAGTGGTGCAGAGAGAAAAAAGAGCAGTGGGAATAGGAGCTTTGTTCCTTAGGTTCTTGGGAGCAGCAGGAAGCACTATGGGCGCAGCCTCAATGACGCTGACGGTACAGGCCAGACAATTATTGTC |
| Subtype C *psi* + single mutation position 13 *env* | CCCACTGCTTAAGCCTCAATAAAGCTTGCCTTGAGTGCTTCAAGTAGTGTGTGCCCGTCTGTTGTGTGACTCTGGTAACTAGAGATCCCTCAGACCAGAAGAGTGGTGCAGAGAGAAAAAAGAGCAGTGGGAATAGGAGCTTTGTTCCTTGGGTTCTTAGGAGCAGCAGGAAGCACTATGGGCGCAGCCTCAATGACGCTGACGGTACAGGCCAGACAATTATTGTC |
| Subtype C *psi* + double mutation (hypermutated) *env* | CCCACTGCTTAAGCCTCAATAAAGCTTGCCTTGAGTGCTTCAAGTAGTGTGTGCCCGTCTGTTGTGTGACTCTGGTAACTAGAGATCCCTCAGACCAGAAGAGTGGTGCAGAGAGAAAAAAGAGCAGTGGGAATAGGAGCTTTGTTCCTTAGGTTCTTAGGAGCAGCAGGAAGCACTATGGGCGCAGCCTCAATGACGCTGACGGTACAGGCCAGACAATTATTGTC |

**Table S2. In silico analysis of subtype B and subtype C sequences for the original and subtype B&C IPDA.** 697 subtype C and 752 Subtype B sequences were subdivided in intact, hypermutated, cis-acting, and “other defective” sequences according to their annotation after sequencing. Other defective sequences included sequences with large deletions, inversions, stop codons, insertions or deletions in essential genes or frame shifts. Psi and env primers and probes were predicted for their annealing towards the specific sequences. The primers and probes of the original assay are depicted as green, the primers and probes of new IPDA are depicted in blue.

|  | ***psi*** | ***env*** | **Indicated result IPDA** |
| --- | --- | --- | --- |
| **Subtype B (n=752/2125)** |  |  |  |
| Intact B&C (n=153) | 100% | 100% | Intact: 100%  Defective: 0%  Inferred lack of signal: 0% |
| Intact Bruner (n=153) | 85.6% | 100% | Intact: 85.6%  Defective: 14.3%  Inferred lack of signal: 0% |
| Hypermutated B&C (n=210) | 88.1% | 2.4% | Intact: 1.9%  Defective: 86.7%  Inferred lack of signal: 11.4% |
| Hypermutated Bruner (n=210) | 59% | 2.4% | Intact: 1%  Defective: 59%  Inferred lack of signal: 40% |
| Other defective B&C (n=288/1661) | 69.4% | 29.5% | Intact: 13%  Defective: 73%  Inferred lack of signal: 14% |
| Other defective Bruner (n=288/1661) | 58.3% | 29.5% | Intact: 13%  Defective: 63%  Inferred lack of signal: 24% |
| Cisacting B&C (n=101) | 39.6% | 100% | Intact: 39%  Defective: 61%  Inferred lack of signal: 0% |
| Cisacting Bruner (n=101) | 2% | 100% | Intact: 2%  Defective: 98%  Inferred lack of signal: 0% |
|  | ***psi*** | ***env*** | **Indicated result IPDA** |
| **Subtype C (n=697)** |  |  |  |
| Intact B&C (n=239) | 100% | 85.8% | Intact: 85.8%  Defective: 14.2%  Inferred lack of signal: 0% |
| Intact Bruner (n=239) | 11.7% | 85.8% | Intact: 11.7%  Defective: 74.1%  Inferred lack of signal: 14.2% |
| Hypermutated B&C (n=89) | 88.8% | 3.4% | Intact: 2.2%  Defective: 87.6%  Inferred lack of signal: 10.1% |
| Hypermutated Bruner (n=89) | 18.0% | 3.4% | Intact: 0%  Defective: 21.3%  Inferred lack of signal: 78.7% |
| Other defective B&C (n=369) | 58.8% | 26.8% | Intact: 10%  Defective: 66%  Inferred lack of signal: 24% |
| Other defective Bruner (n=369) | 9.2% | 26.8% | Intact: 2%  Defective: 32%  Inferred lack of signal: 66% |

**Figure S1. Overview of the pipelines to classify intact sequences for both subtypes.** Sequences were classified by a process of elimination of sequences with multiple defects in the different subtype B or subtype C pipelines. The sequences which did not show any defects were classified as intact. ORF= Open reading Frame, MSD = Major Splice Donor.

**Figure S2. Detection efficiency of different subtype primers.** The median values with 95% confidence interval of 4 replicates of equal Gblock copies for the specific subtypes were run for the two different primer subtype sets. No significant differences (unpaired T-test) were found between the different primers (p>0.6). Data is shown for an annealing temperature of 60˚C, identical results were found for an annealing temperature of 55˚C.

**Figure S3. Calculation example.** Copy numbers for both RPP30 and *psi/env* are examples of numbers that can be found in clinical samples. The subsequent calculations for the shearing ratio, number of cells in the assay and the HIV copy numbers per million cells are given for two situations: plenty (above) and limited (below in red) number of HIV copies.
